# Surgically induced brain‐draining lymphatic dysfunction accelerates the development of cognitive impairment

**DOI:** 10.1002/alz70856_096732

**Published:** 2025-12-24

**Authors:** Jiaqi Sun, Alanna Spiteri, Xin Huang, Colin L. Masters, Liang Jin, Yijun Pan

**Affiliations:** ^1^ University of Melbourne, Parkville, VIC, Australia; ^2^ Florey Institute of Neuroscience and Mental Health, Parkville, VIC, Australia; ^3^ The Florey Institute of Neuroscience and Mental Health, Parkville, VIC, Australia; ^4^ Florey Institute of Neuroscience and Mental Health, Melbourne, VIC, Australia

## Abstract

**Background:**

Mounting evidence suggests the involvement of the lymphatic system in the pathogenesis of Alzheimer's disease (AD). In this study, we examined the impact of brain‐draining lymphatic dysfunction on the cognition of wildtype (WT) and transgenic (TG) APP/PS1 mice.

**Method:**

Deep cervical lymph ducts (dcLNs) ligation or sham surgery was performed on 3‐month‐old TG mice and their WT littermates. Brain lymphatic drainage dysfunction was confirmed using fluorescent beads that were injected into the cisterna magna, and the dcLNs were harvested one hour after injection for fluorescence bead counting. The sham‐operated mice underwent exposure of the dcLNs without ligation. One month later, cognition was assessed using the T‐maze spontaneous alternation test, and Y‐maze short‐term spatial learning and memory test.

**Result:**

A significant reduction in fluorescence bead drainage to the dcLNs was observed in the brain‐draining lymphatics‐ligated mice compared to sham‐operated mice (one‐sample Wilcoxon test, *p* = 0.016). For the T maze, two‐way ANOVA revealed a main effect of ligation (F_1,46_ = 4.420, *p* =  0.04), indicating that impaired brain‐draining lymphatics compromised working memory, regardless of genotype. For the Y maze, surgically induced brain‐draining lymphatics dysfunction accelerated the development of short‐term spatial learning and memory deficits. Sham‐operated WT and TG mice were able to differentiate the novel arm from familiar arm (adj *p* =  0.0009, *p* =  0.015); however, brain‐draining lymphatics ligated WT and TG mice failed to do so (adj *p* =  0.50, *p* =  0.32).

**Conclusion:**

These findings suggest that impaired brain‐draining lymphatics contribute to the development of cognitive impairment in both WT and TG APP/PS1 mice. Future studies should address the underlying mechanisms responsible for this observation.

